# Supplementary material for: Endothelial cell clonal expansion in the development of cerebral cavernous malformations
Source: Nat Commun. 2019 Jun 24;10:2761. doi: 10.1038/s41467-019-10707-x (PMC6591323; doi:10.1038/s41467-019-10707-x)
Supplement: Supplementary file 1 — Supplementary Information [file 41467_2019_10707_MOESM1_ESM.pdf]

Endothelial cell clonal expansion

in the development of Cerebral Cavernous Malformations

Malinverno et al.

Supplementary Information

Supplementary Figure 1

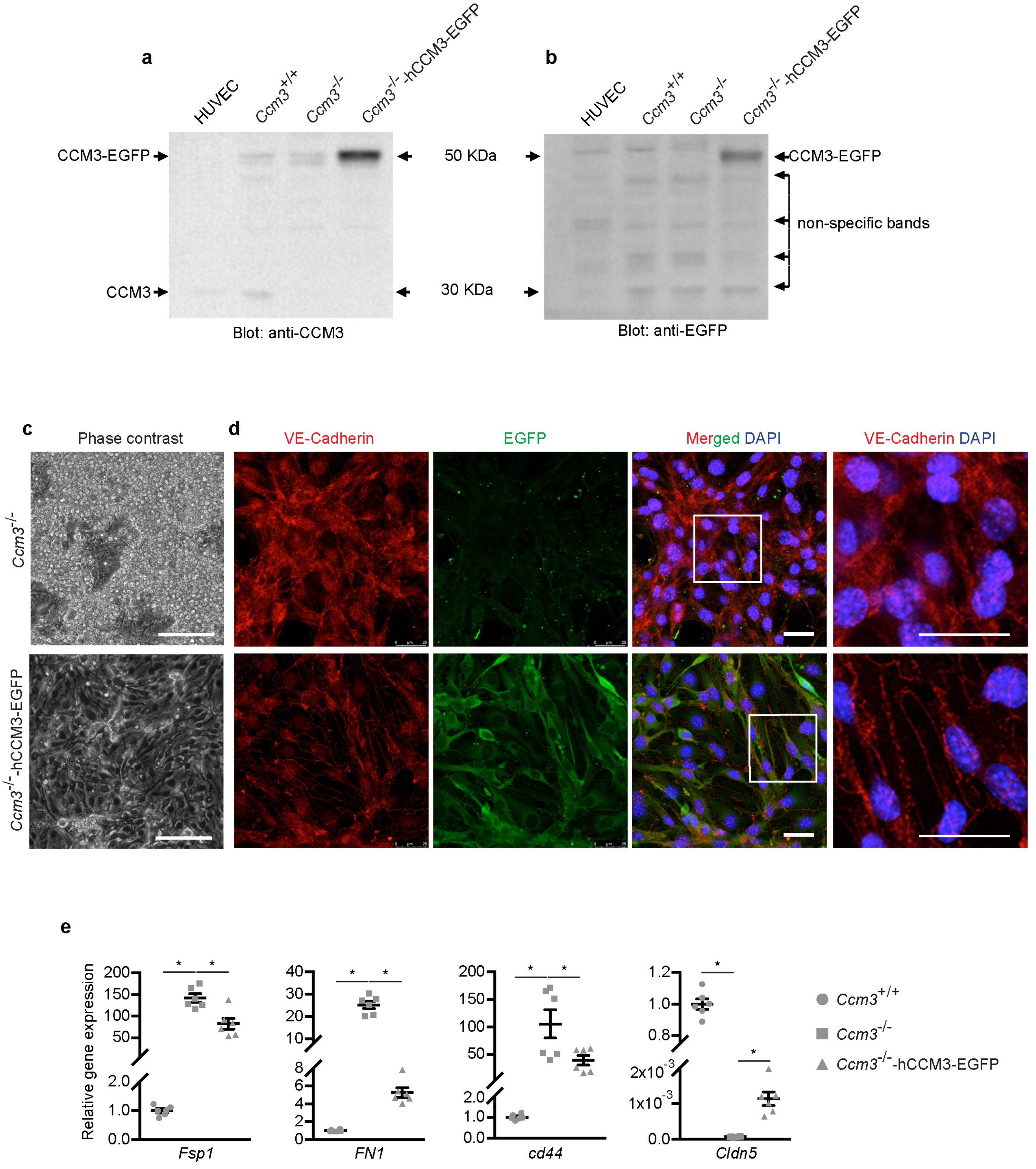

Supplementary Figure 1. Rescue experiment on *Ccm3*$^{-/-}$ cells

Immortalised lung *Ccm3*$^{-/-}$ endothelial cells were infected with a lentiviral vector to express the human CCM3 fused to the EGFP. **(a,b)** Representative Western Blot from total lysate of HUVEC, *Ccm3*$^{+/+}$, *Ccm3*$^{-/-}$ and reconstituted *Ccm3*$^{-/-}$ murine cells (*Ccm3*$^{-/-}$ -hCCM3-EGFP). Membranes were blotted for CCM3 **(a)** or EGFP **(b)** and show the ablation of endogenous protein in *Ccm3*$^{-/-}$ cells and the re-expression of the CCM3-EGFP in *Ccm3*$^{-/-}$ -hCCM3-EGFP cells. The molecular weight of the fusion protein CCM3-EGFP is around 36 kDa higher than the endogenous CCM3 protein. **(c)** Representative phase contrast image showing the morphology of *Ccm3*$^{-/-}$ and *Ccm3*$^{-/-}$ -hCCM3-EGFP cells. **(d)** Representative confocal images of *Ccm3*$^{-/-}$ and *Ccm3*$^{-/-}$ -hCCM3-EGFP cells stained for VE-Cadherin (red) and EGFP (green) to show the re-organization of adherens junctions upon re-expression of human CCM3. Nuclei are counterstained with DAPI. Scale bars, 250 μm **(a)**, 25 μm **(d)**. **(e)** RT-qPCR analysis on *Ccm3*$^{+/+}$, *Ccm3*$^{-/-}$ *Ccm3*$^{-/-}$ -hCCM3-EGFP cells. Data are means ± SE. $p < 0.01$ among groups (one-way ANOVA); * $p < 0.01$ (Tukey's post-hoc test); n = 6 independent samples. Source data are provided as a Source Data file.

Supplementary Figure 2

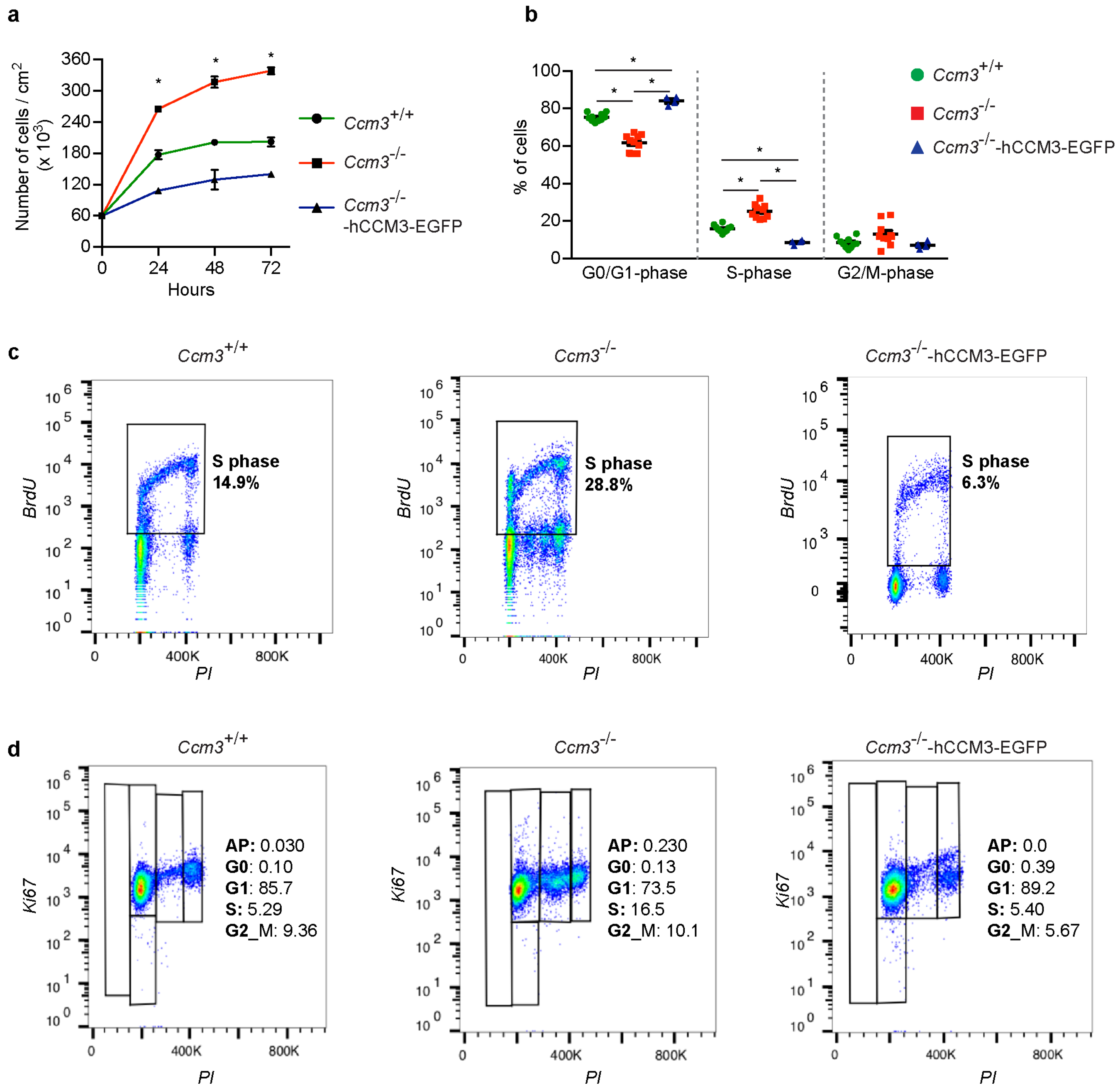

Supplementary Figure 2. *Ccm3* regulates proliferation and cell cycle in endothelial cells.

(a) Growth curve performed with *Ccm3*$^{+/+}$, *Ccm3*$^{-/-}$ and reconstituted *Ccm3*$^{-/-}$ -hCCM3-EGFP immortalised lung endothelial cells. Cells were plated at 60'000 cells/cm^2 and then harvested and counted after 24, 48 and 72 hours. *Ccm3*$^{-/-}$ cells were more proliferative than the *Ccm3*$^{+/+}$, while the re-expression of hCCM3 completely rescued the phenotype. Moreover, *Ccm3*$^{+/+}$ stopped growing after having gained the plateau confluency at 48 hours, while the Ccm3$^{-/-}$ cells did not show contact inhibition of growth. Data are means ± SE; n = 3 independent experiments; p <0.01 among groups (two-ways ANOVA); * p <0.01 within each time point with Tukey's test. **(b)** Plot showing the distribution *Ccm3*$^{+/+}$, *Ccm3*$^{-/-}$ and reconstituted *Ccm3*$^{-/-}$ -hCCM3-EGFP cells among the phases of the cell cycle as assessed by FACS analysis with the fluorescent intercalating agent Propidium Iodide (PI). *Ccm3*$^{-/-}$ cells were dividing more frequently than the two counterparts, as the percentage of *Ccm3*$^{-/-}$ cells in the S-phase is higher if compared to the wild-type and the reconstituted conditions. Data are means ± SE. p <0.01 among groups (one-way ANOVA); * p <0.01 (Tukey's post-hoc test); n = 10 independent samples for *Ccm3*$^{+/+}$and *Ccm3*$^{-/-}$ cells, n = 4 independent samples for *Ccm3*$^{-/-}$ - hCCM3-EGFP . Source data are provided as a Source Data file. **(c)** Cells were incubated with a thymidine analogue, the 5'-bromo-2'-deoxyuridine (BrdU) for 3 hours. Cells that have incorporated BrdU has further been stained with anti-BrdU antibodies and fluorescent DNA dye (PI) to separate the cells according to the cell cycle phases (i.e., G1, S, G2/M phases). The plot shows the increased percentage of cells in S-phase in the *Ccm3*$^{-/-}$ cells, compared to *Ccm3*$^{+/+}$ cells, and the rescue after re-expression of hCCM3. **(d)** FACS analysis of cells labelled with PI and for the proliferative marker Ki67, dissect their distribution among the cell cycle phases. The sequential transition between G1-S-G2-M phases resulted to be remarkably different between the wild-type, the knock-out and the reconstituted condition. In fact, the percentage of *Ccm3*$^{-/-}$ cells in both S-phase and G2/M-phase is higher compared to the *Ccm3*$^{+/+}$ and the reconstituted *Ccm3*$^{-/-}$ cells, confirming the altered proliferative feature of the *Ccm3*$^{-/-}$ cells.

See also **Supplementary Figure 11** for the gating strategy.

Supplementary Figure 3

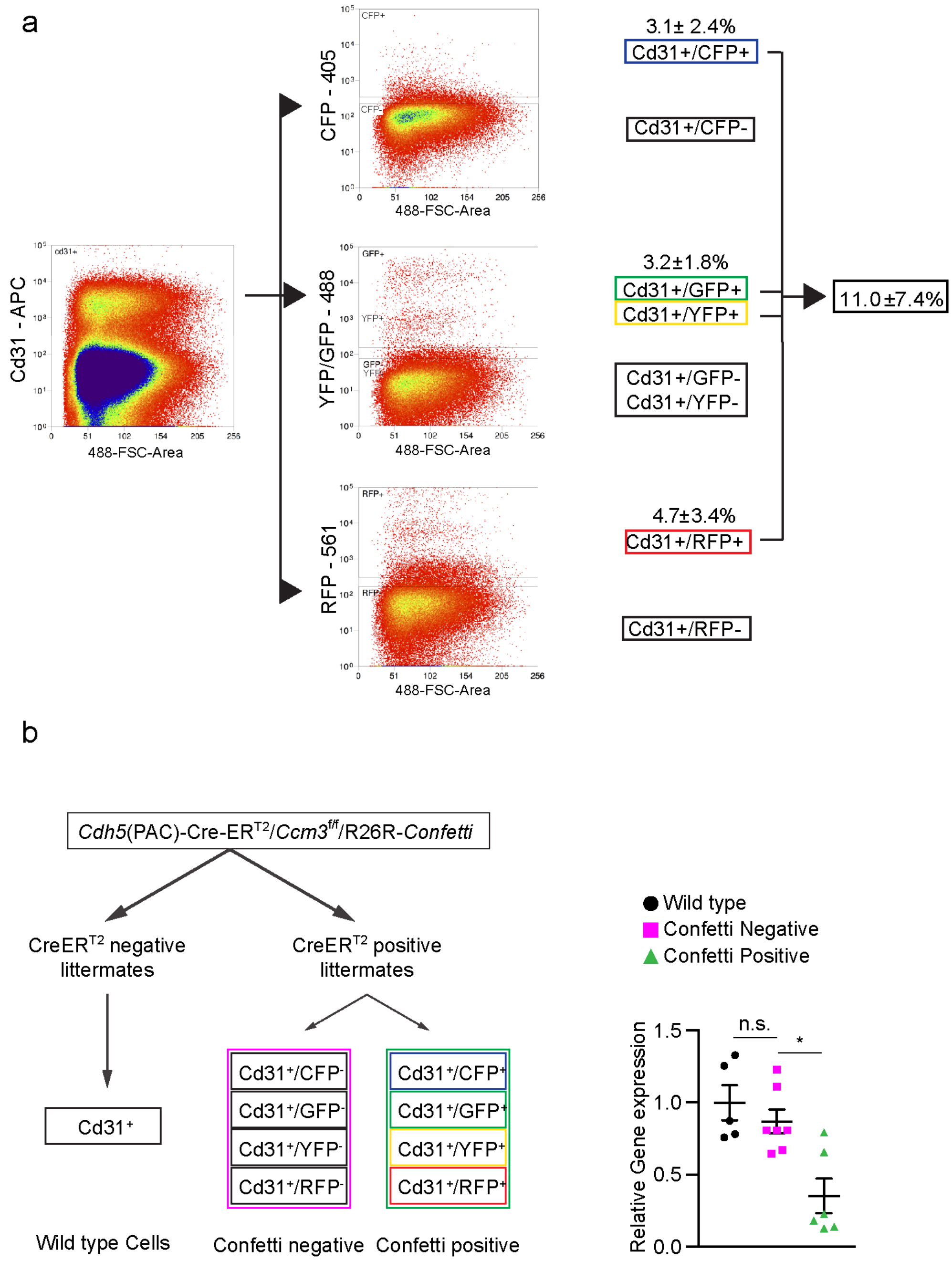

Supplementary Figure 3. *Confetti*-positive endothelial cells show recombination for *Ccm3*

Endothelial cells isolated from the chronic *Cdh5(PAC)*-Cre-ER^{T2}/*Ccm3*$^{f/f}$/R26R-*Confetti* mouse model were sorted by FACS and analysed for *Ccm3* expression. **(a)** Endothelial cells were labelled with an anti-Cd31-APC antibody, and then sorted on the basis of expression of the four *Confetti* fluorescent proteins: CFP, GFP, YFP and RFP. GFP and YFP could not be separated, and so were considered together. Then, $Cd31^+/CFP^+$, $Cd31^+/GFP^+/YFP^+$ and $Cd31^+/RFP^+$ cells were pooled as *Confetti* positive (11.0% ±7.4%), while $Cd31^+/CFP^-$, $Cd31^+/GFP^-/YFP^-$ and $Cd31^+/RFP^-$ cells were pooled as *Confetti* negative. Data are means ±SE. n = 5 independent experiments. See also **Supplementary Figure 11** for the gating strategy. **(b)** *Confetti*-positive and -negative endothelial cells were isolated as described above. In parallel, Cre-ER^{T2}-negative littermates were used as wild-type controls to isolate endothelial cells with no recombination, after labelling with an anti-Cd31-APC antibody. *Ccm3* expression was analysed by RT-qPCR on these wild-type, *Confetti*-negative and *Confetti*-positive endothelial cells. Data are means ±SE. $p < 0.01$ among groups (one-way ANOVA); * $p < 0.004$, n.s. $p = 0.377$ (Tukey's post-hoc test); n = 5 independent experiments. Source data are provided as a Source Data file.

Supplementary Figure 4

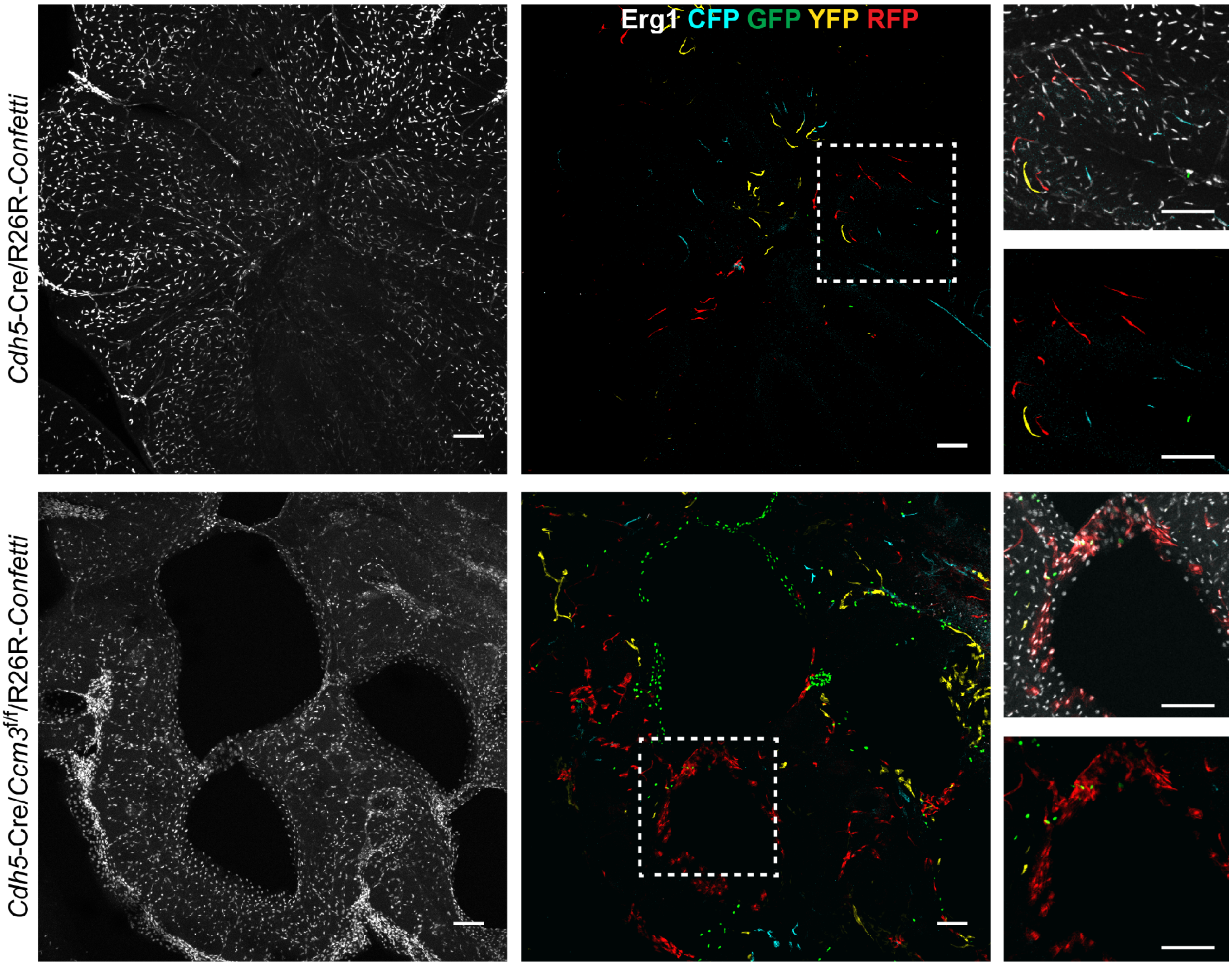

Supplementary Figure 4. Clonal expansion in *Cdh5(PAC)*-Cre-ER^{T2}/*Ccm3*$^{f/f}$/R26R-*Confetti* mice

Representative confocal images of chronic *Cdh5(PAC)*-Cre-ER^{T2}/R26R-*Confetti* or *Cdh5(PAC)*-Cre-ER^{T2}/*Ccm3*$^{f/f}$/R26R-*Confetti* mice analysed at P30, showing the clonal expansion of endothelial cells within the lesions in comparison to normal wild type vessels. Nuclei of endothelial cells are stained for Erg1. Scale bar 100 µm

Supplementary Figure 5

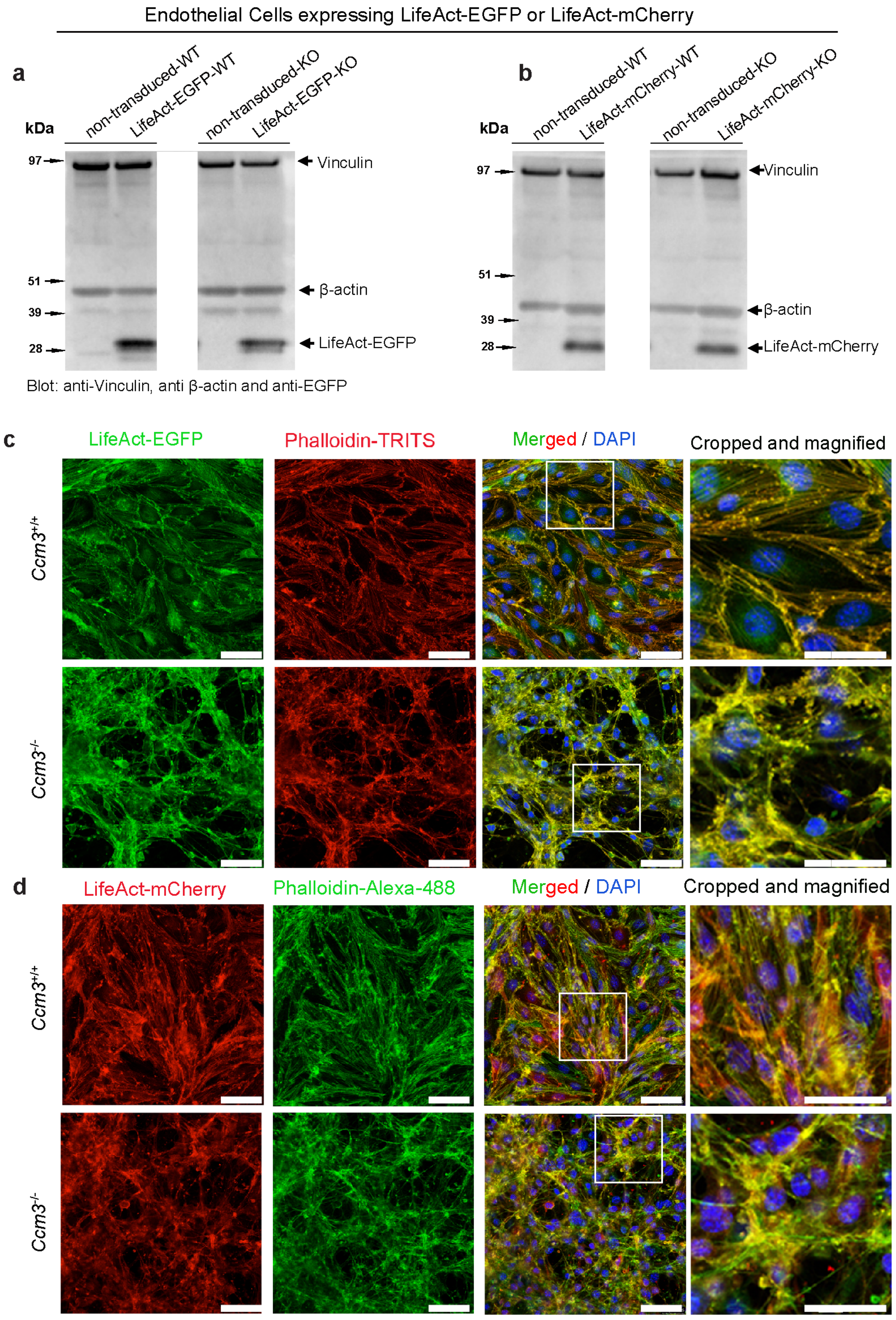

Supplementary Figure 5. Generation of LifeAct-transduced endothelial cell lines

LifeAct-mCherry and LifeAct-EGFP fusions are functionally competent when expressed in immortalised lung endothelial cells. **(a, b)** Representative Western blotting of non-transduced *Ccm3*$^{+/+}$ cells, *Ccm3*$^{+/+}$ cells expressing LifeAct-EGFP, non-transduced *Ccm3*$^{-/-}$ cells, and *Ccm3*$^{-/-}$ cells expressing LifeAct-EGFP **(a)** or LifeAct-mCherry **(b)**. The membranes were probed with anti-vinculin antibody as the loading control, and anti-β-actin antibody, and with either anti-EGFP antibody **(a)** or anti-mCherry antibody **(b)** as indicated. **(c, d)** Representative images of *Ccm3*$^{+/+}$ and *Ccm3*$^{-/-}$ cells expressing either LifeAct-EGFP **(c)** or LifeAct-mCherry **(d)** that were fixed and subsequently labelled with either TRITC-phalloidin or AlexaFluor488-phalloidin. Nuclei were stained using DAPI (blue). Boxes indicate cropped and magnified areas. Scale bars, 50 µm. Source data are provided as a Source Data file.

Supplementary Figure 6

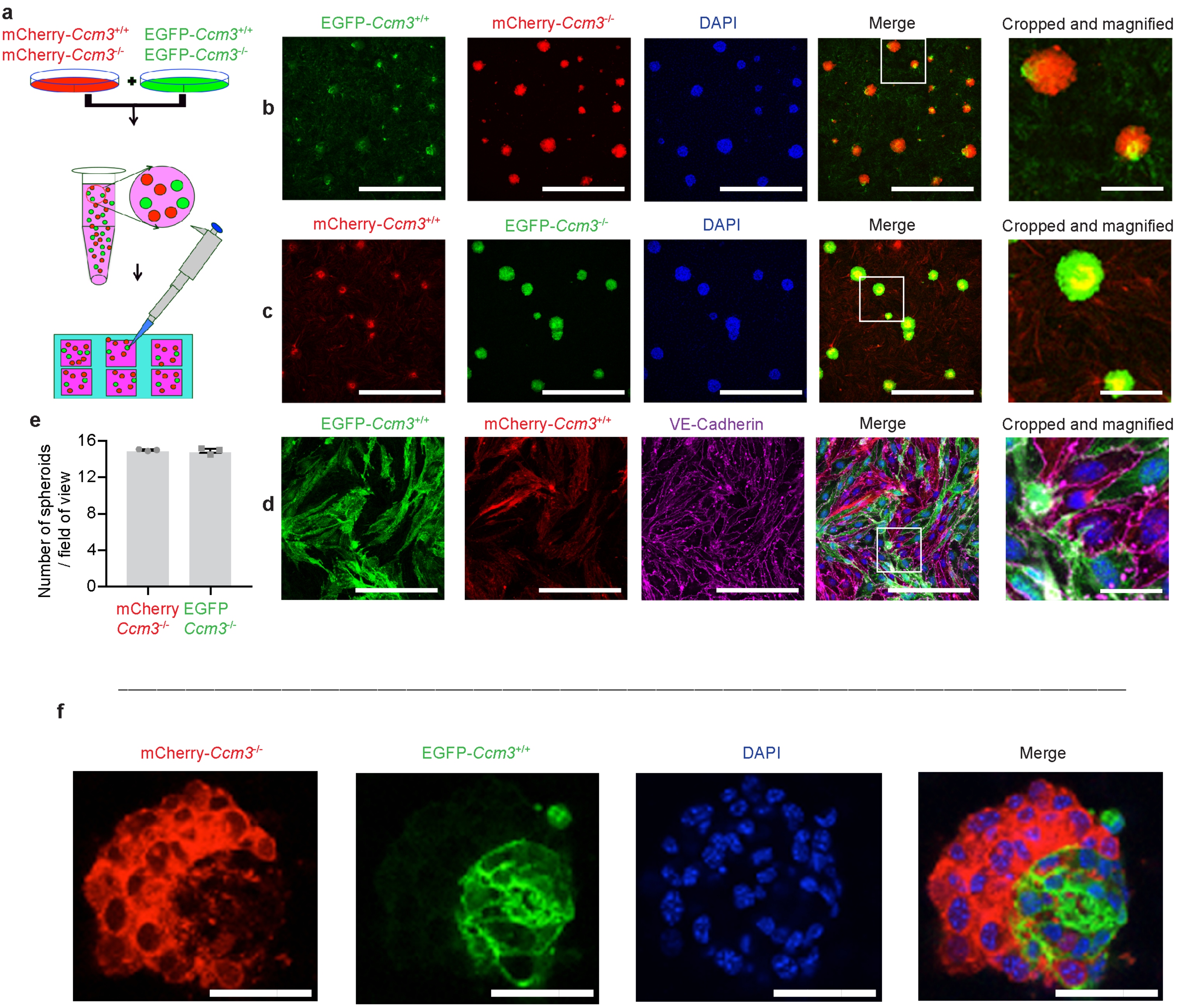

Supplementary Figure 6. Co-culture of *Ccm3*$^{+/+}$ and *Ccm3*$^{-/-}$ endothelial cells

Ccm3$^{-/-}$ cells form spheroids when co-cultured with their *Ccm3*$^{+/+}$ counterparts. **(a)** Scheme of cell seeding. The cells were trypsinised and then co-cultured, with representative images shown for *Ccm3*$^{+/+}$ cells expressing LifeAct-EGFP and *Ccm3*$^{-/-}$ cells expressing LifeAct-mCherry **(b)**, *Ccm3*$^{+/+}$ cells expressing LifeAct-mCherry and *Ccm3*$^{-/-}$ cells expressing LifeAct-EGFP, and **(c)** *Ccm3*$^{+/+}$ cells expressing LifeAct-EGFP and *Ccm3*$^{+/+}$ cells expressing LifeAct-mCherry **(d)**. **(e)** Quantification of the numbers of spheroids formed per field of view. Data are means ± SE; p=0.6675 (Student's t-test); n = 3 independent experiments. **(f)** Representative confocal z-stack images of *Ccm3*$^{-/-}$ cells expressing LifeAct-mCherry and *Ccm3*$^{+/+}$ cells expressing LifeAct-EGFP. Scale bars, 50 μm; 10 μm (magnified). Source data are provided as a Source Data file.

Supplementary Figure 7

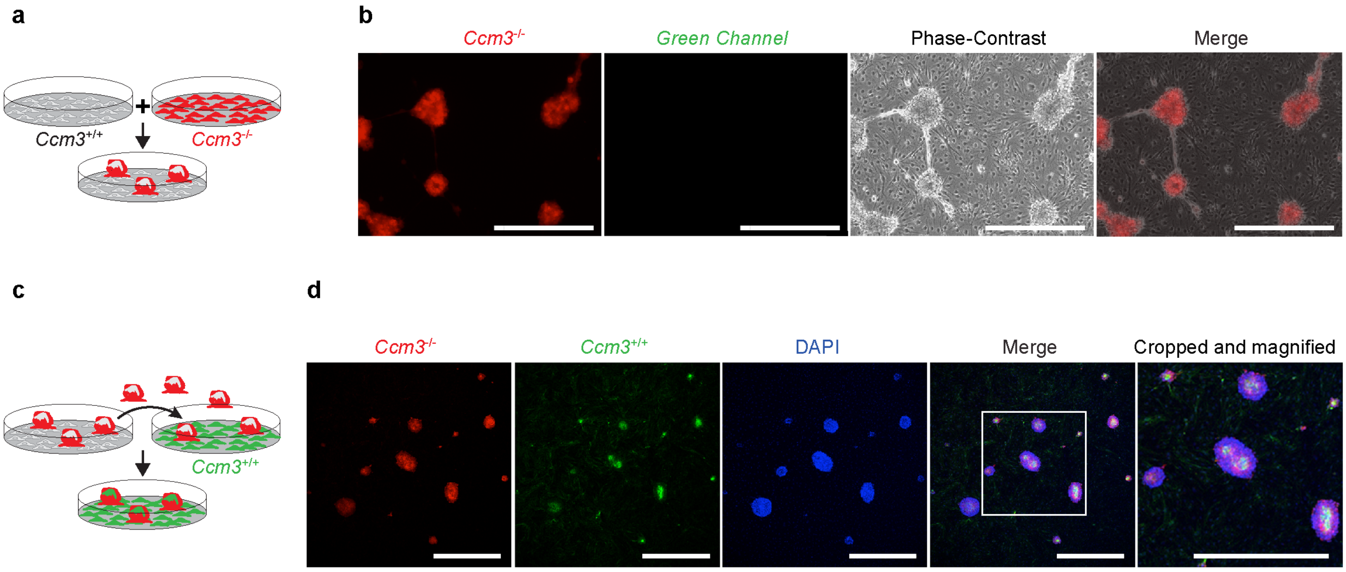

Supplementary Figure 7. ***Ccm3***$^{-/-}$ **spheroids recruit** ***Ccm3***$^{+/+}$ **cells**

(a, b) Non-labelled *Ccm3*$^{+/+}$ cells and LifeAct-mCherry–expressing *Ccm3*$^{-/-}$ cells were co-cultured for 7 days, until spheroid formation. Experimental scheme **(a)** and representative images of spheroid formation **(b)**. **(c, d)** Spheroids were detached by gently shaking the culture plates, and then collected and added to already formed monolayers of LifeAct-EGFP *Ccm3*$^{+/+}$ cells. Experimental scheme **(a)** and representative images of spheroids that have recruited *Ccm3*$^{+/+}$ cells **(b)**. Scale bars, 500 µm.

Supplementary Figure 8

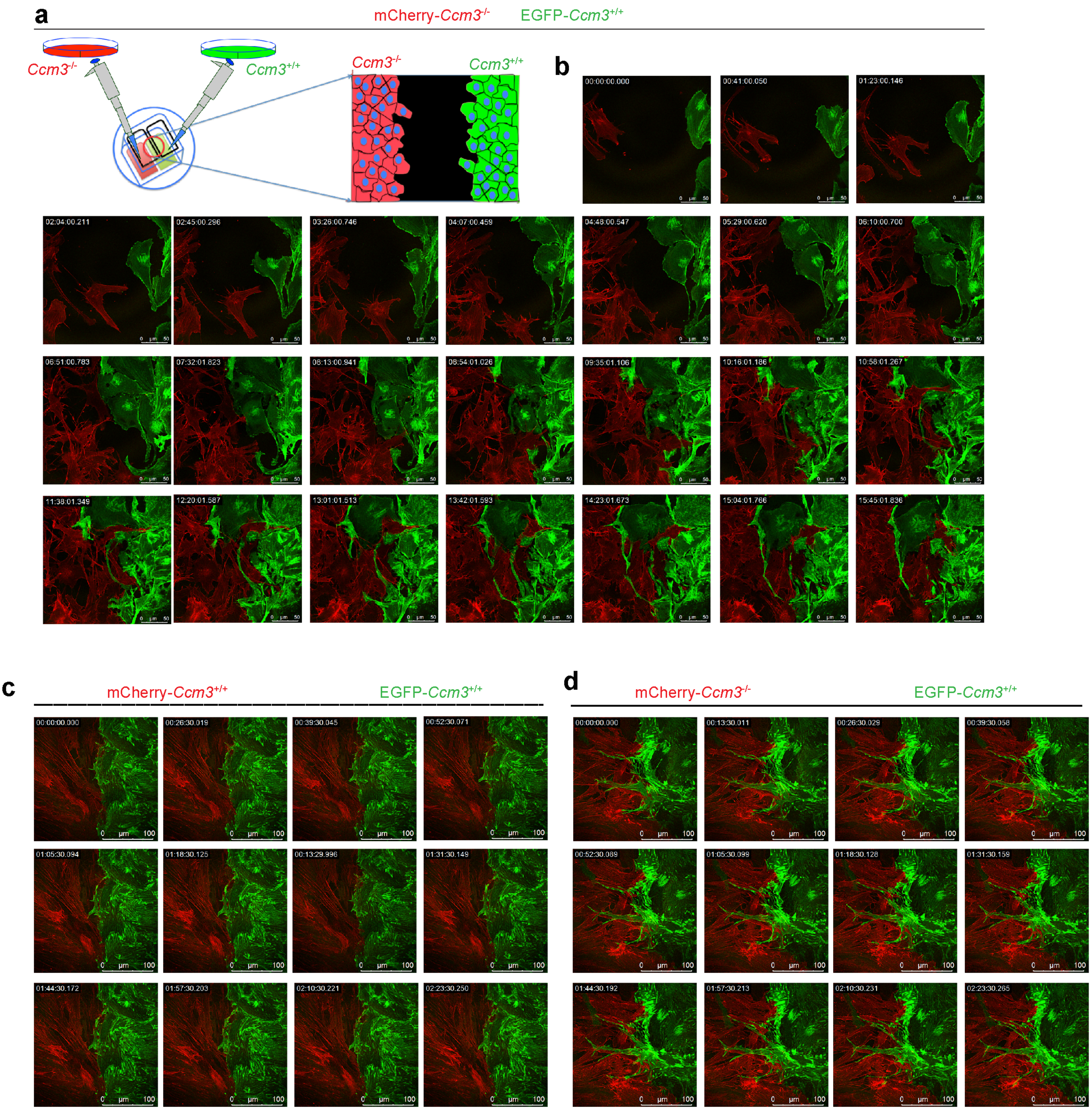

Supplementary Figure 8. Wound-healing assays with *Ccm3*$^{+/+}$ and *Ccm3*$^{-/-}$ endothelial cells

(a) Scheme of cell seeding for mixed wound-healing assays. **(b)** *Ccm3*$^{-/-}$ cells expressing LifeAct-mCherry and *Ccm3*$^{+/+}$ cells expressing LifeAct-EGFP were seeded in each side of ibidi silicon inserts and grown to confluence. After insert removal, time-lapse images (h:min:s:ms) were acquired every 2 min until the cells had closed the gap, with selected images shown at given times . These frames are taken form the **Supplementary Movie 10**. **(c, d**) *Ccm3*$^{+/+}$ cells expressing LifeAct-mCherry and LifeAct-EGFP **(c)** and *Ccm3*$^{-/-}$ cells expressing LifeAct-mCherry and *Ccm3*$^{+/+}$ cells expressing LifeAct-EGFP **(d)** were seeded in each side of ibidi silicon inserts and grown to confluence. After insert removal the cells were left to close the gap, and once they had made contact, time-lapse images (h:min:s:ms) were acquired each 2 min, with selected images shown at given times. *Ccm3*$^{+/+}$ cells started to extend large protrusions and to invade only the first row of the *Ccm3*$^{-/-}$ cell layer, but did not pass into the middle of the culture; these structures developed only in the presence of the *Ccm3*$^{-/-}$ cells, and not with the *Ccm3*$^{+/+}$ cells on the other side of the wound, which indicated that the *Ccm3*$^{-/-}$ cells can attract the *Ccm3*$^{+/+}$ cells. These frames are taken form the **Supplementary Movie 11.**

Supplementary Figure 9

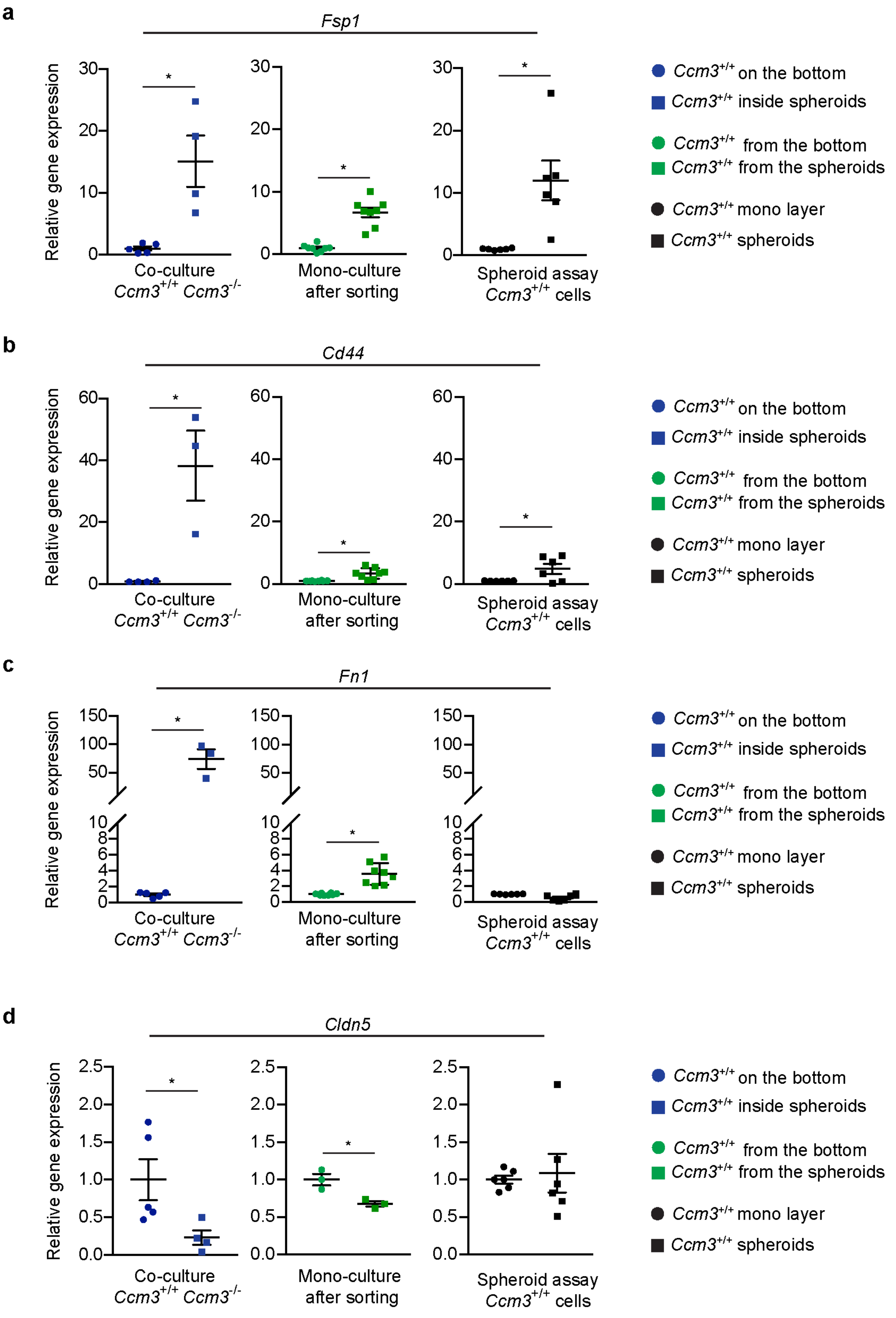

Supplementary Figure 9. Induction of EndMT is reversible and not due to 3D culture

(a-d) RT-qPCR analysis for the genes *Fsp1*, *Cd44*, *Fn1* and *Claudin5*. Data are means ± SE; * $p < 0.05$ (Student's t-test); each dot represents an independent sample.

For each gene, the plot shows the values of:

- *Ccm3*$^{+/+}$ cells isolated from the co-culture, as reported in Figure 7g, for reference **(left)**;
- *Ccm3*$^{+/+}$ cells isolated after co-culture from either the spheroids or the bottom and kept in culture separately **(centre)**;
- *Ccm3*$^{+/+}$ cells cultured alone as monolayer or spheroids on methylcellulose **(right).**

Source data are provided as a Source Data file.

Supplementary Figure 10

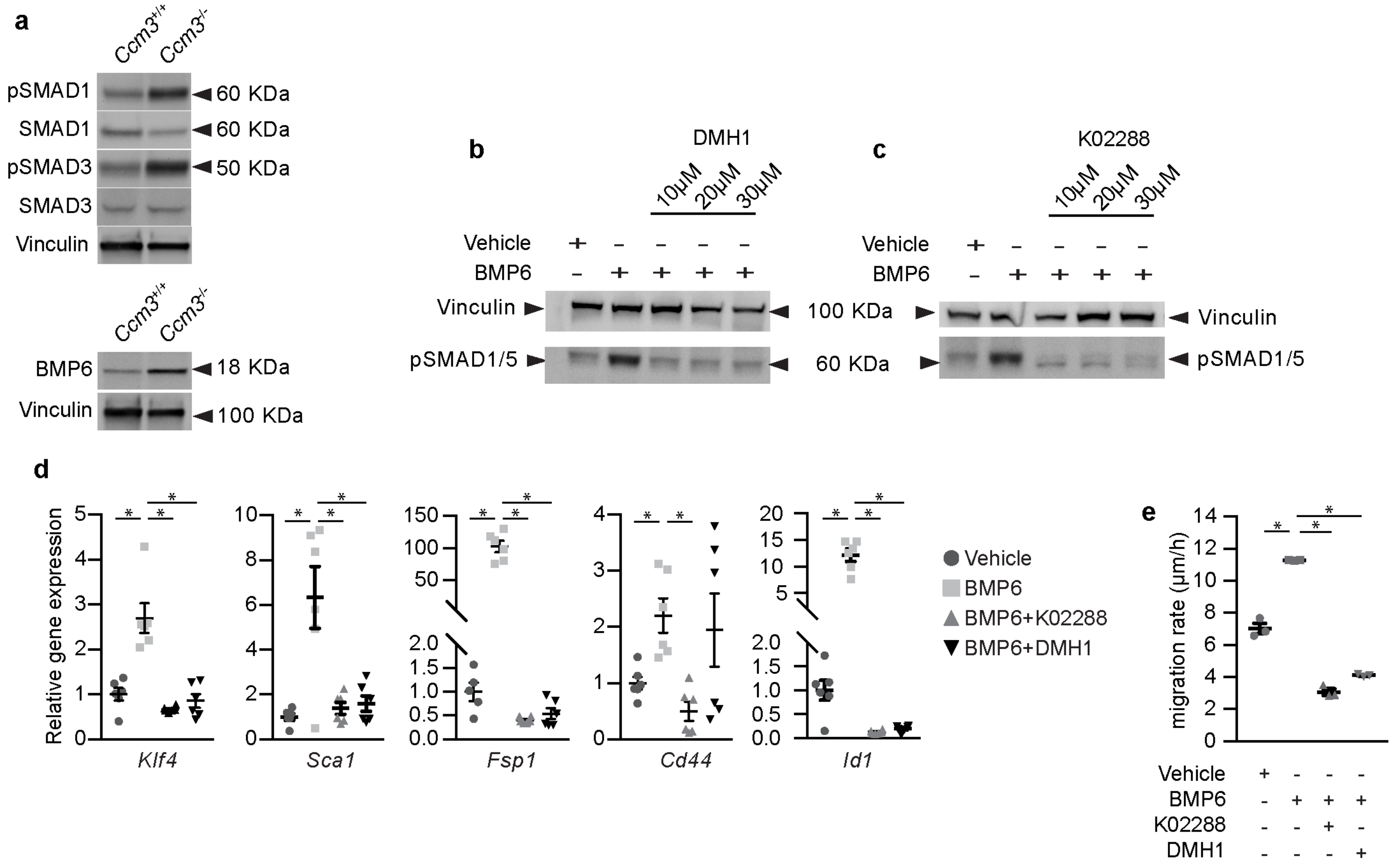

Supplementary Figure 10. The BMP pathway is involved in the induction of migration and EndMT in *Ccm3*$^{+/+}$ endothelial cells.

(a-c) Representative Western blotting of total lysate from *Ccm3*$^{+/+}$ and *Ccm3*$^{-/-}$ endothelial cells **(a)**, and of *Ccm3*$^{+/+}$ endothelial cells pre-incubated for 1 h with either DMH1 **(b)** or K02288 **(c)** at the indicated concentrations, and then stimulated with 100 ng/ml BMP6 for 1 h. Both K02288[53] and DMH1[36,39] are inhibitors of the BMP6 pathway. **(d)** Quantification of relative gene expression of *Ccm3*$^{+/+}$ endothelial cells either pre-incubated for 1 h with 10 µM DMH1 or 10 µM K02288 and then stimulated for 6 days with 100 ng/ml BMP6. Data are means ±SE; $p < 0.02$ among groups (one-way ANOVA); * $p < 0.01$ (Tukey's post-hoc test); n = 6 independent samples. **(e)** Quantification of migration rate of *Ccm3*$^{+/+}$ endothelial cells pre-incubated for 1 h without or with 10 µM DMH1 or 10 µM K02288 and then stimulated with 100 ng/ml BMP6. After stimulation, a wound was made and the migration rates for the healing were measured after 48 h. Data are means ±SE. $p < 0.001$ among groups (one-way ANOVA); * $p < 0.01$ (Tukey's post-hoc test); n = 3 independent samples. Source data are provided as Source Data file.

Supplementary Figure 11

ns

a

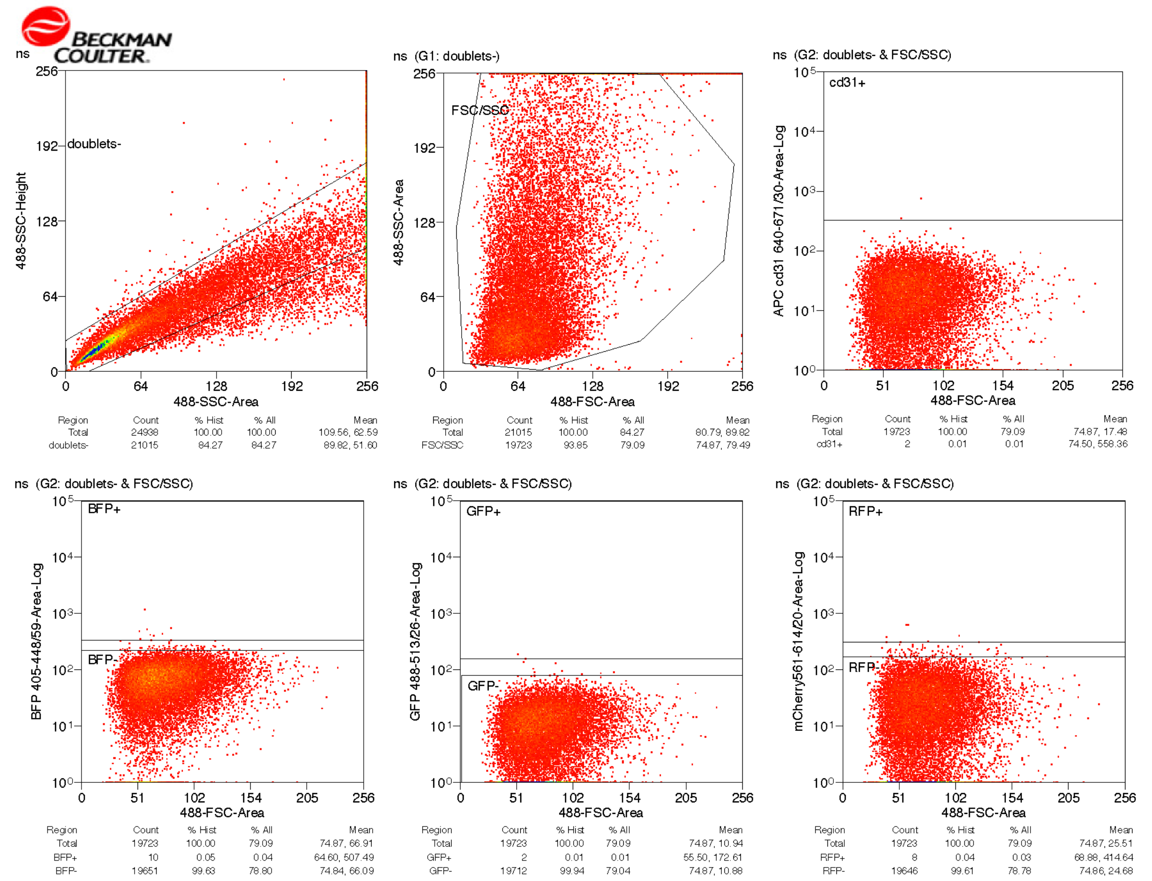

conf1

Thursday, March 09, 2017

b

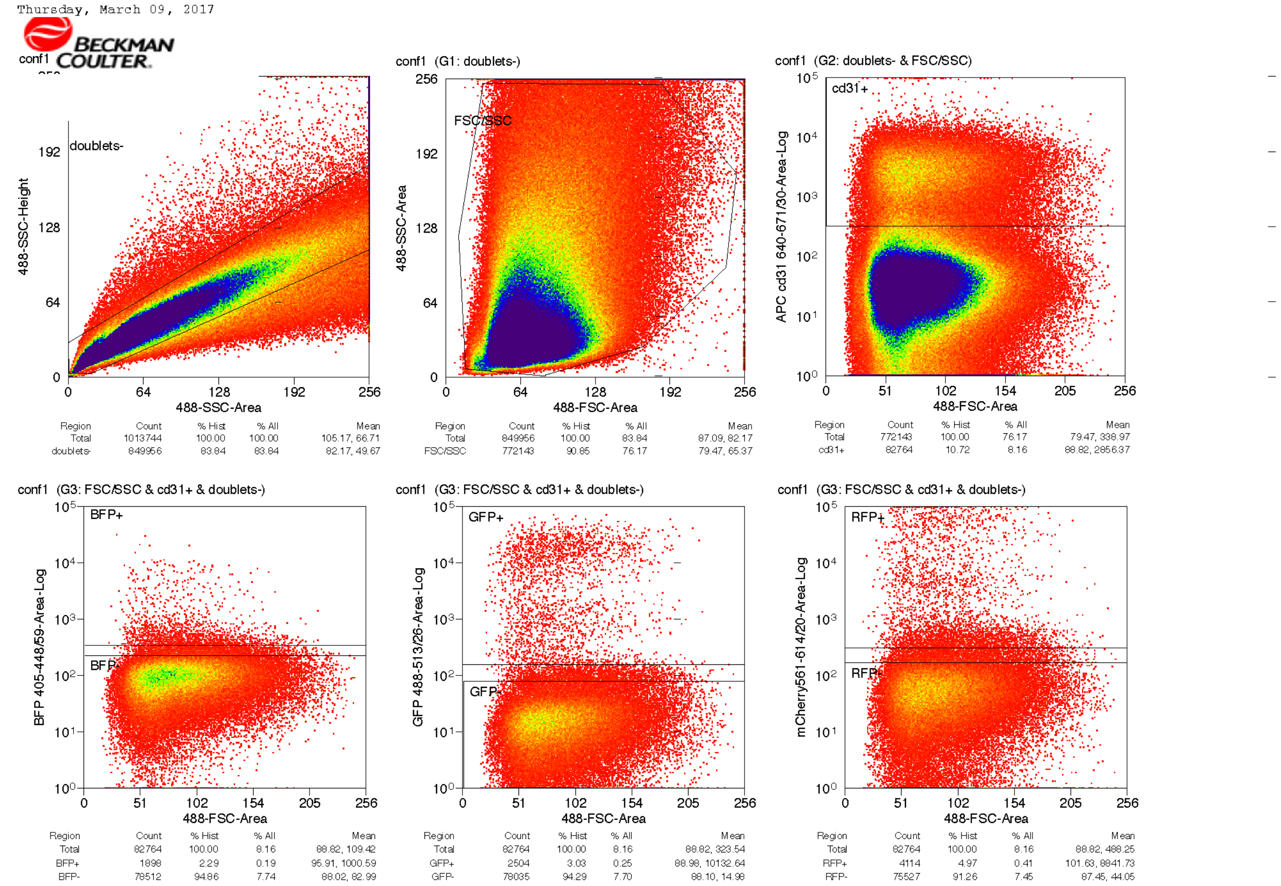

Thursday, March 09, 2017

Supplementary Figure 11. Gating strategy for sorting of Cd31^{+}/*Confetti*$^{+}$ cells from *Confetti* reporter mice.

Representative scheme of gating strategy for the isolation of Cd31^{+}/Confetti^{+} endothelial cells from mouse brain.

a) Gated have been set on cells isolated from a *Confetti* negative mouse not stained for Cd31.

b) Cells from *Confetti* positive mice which have undergone recombination, were labelled with APC conjugated anti Cd31 antibody. After gating for Cd31, endothelial cells were around 8% of total cells.

Supplementary Figure 12

a

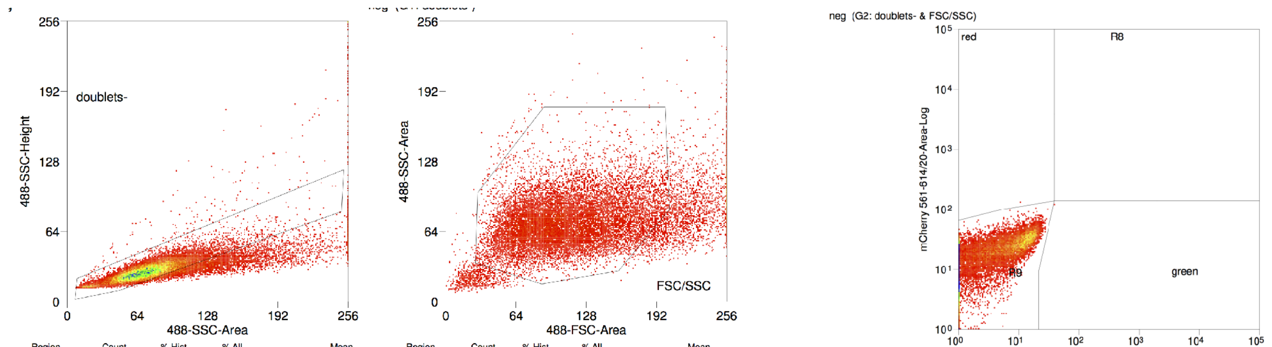

Region	Count	% Hist	% All	Mean
Total	23442	100.00	100.00	87.21, 30.23
doublets-	19180	81.82	81.82	73.22, 27.91

Region	Count	% Hist	% All	Mean
Total	19180	100.00	81.82	122.75, 73.22
FSC/SSC	15734	82.03	67.12	109.61, 72.68

Region	Count	% Hist	% All	Mean
Total	15734	100.00	67.12	6.01, 21.64
red	1	0.01	0.00	10.46, 131.11
R8	0	0.00	0.00	0.00, 0.00
R9	15732	99.99	67.11	6.01, 21.63
green	1	0.01	0.00	38.75, 119.79

b

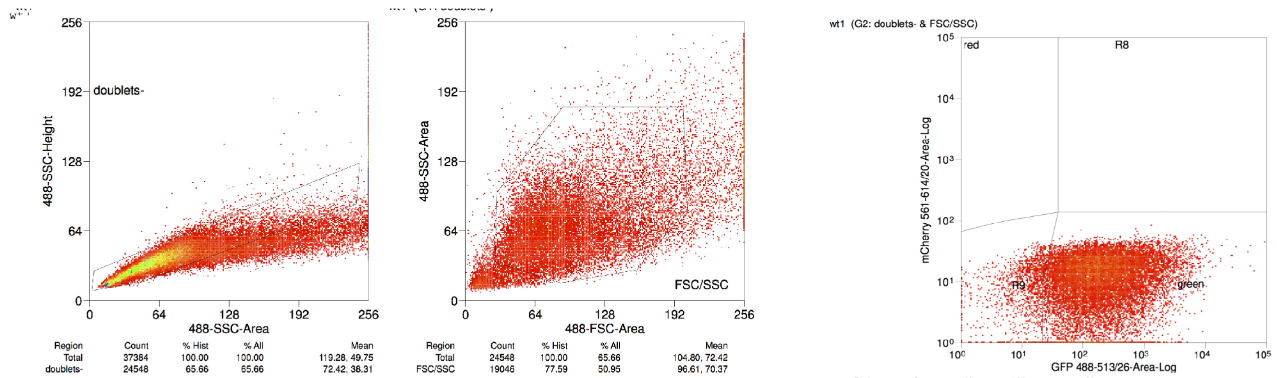

Region	Count	% Hist	% All	Mean
Total	37384	100.00	100.00	119.28, 49.75
doublets-	24548	65.66	65.66	72.42, 38.31

Region	Count	% Hist	% All	Mean
Total	24548	100.00	65.66	104.80, 72.42
FSC/SSC	19046	77.59	50.95	96.61, 70.37

Region	Count	% Hist	% All	Mean
Total	19046	100.00	50.95	471.68, 14.24
red	0	0.00	0.00	0.00, 0.00
R8	0	0.00	0.00	0.00, 0.00
R9	1442	7.57	3.86	12.69, 11.84
green	17604	92.43	47.09	509.26, 14.43

c

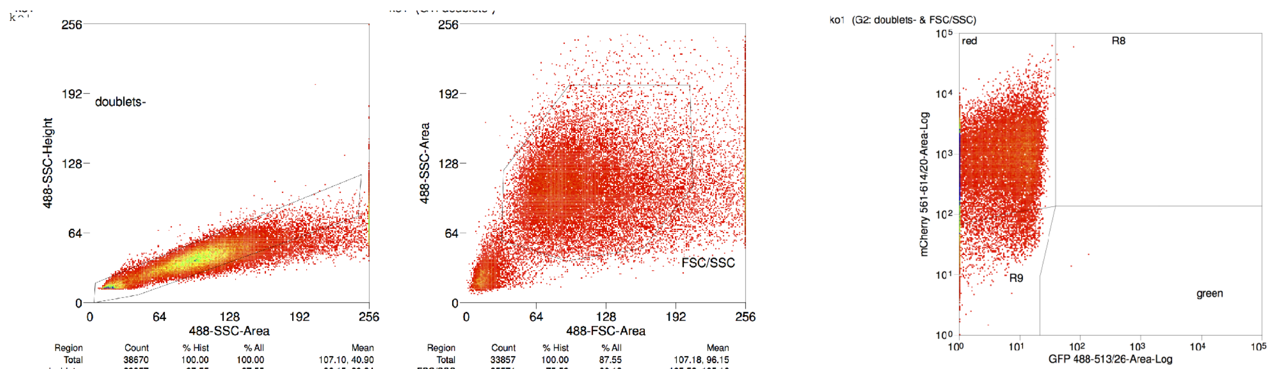

Region	Count	% Hist	% All	Mean
Total	38670	100.00	100.00	107.10, 40.90
doublets-	33857	87.55	87.55	96.15, 39.24

Region	Count	% Hist	% All	Mean
Total	33857	100.00	87.55	107.18, 96.15
FSC/SSC	25571	75.53	66.13	105.52, 105.16

Region	Count	% Hist	% All	Mean
Total	25571	100.00	66.13	6.56, 1394.13
red	22905	89.57	59.23	6.60, 1542.17
R8	4	0.02	0.01	49.94, 47681.40
R9	2660	10.40	6.88	6.11, 50.80
green	2	0.01	0.01	103.43, 17.63

d

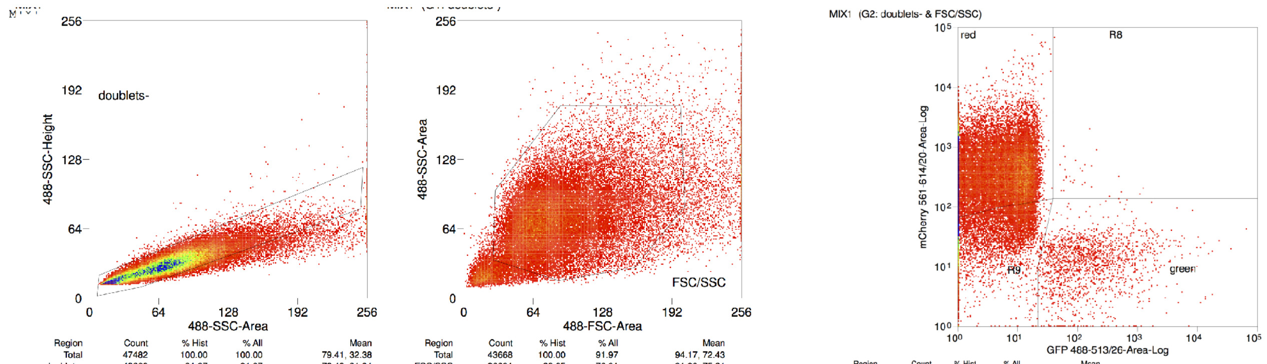

Region	Count	% Hist	% All	Mean
Total	47482	100.00	100.00	79.41, 32.38
doublets-	43668	91.97	91.97	72.43, 31.04

Region	Count	% Hist	% All	Mean
Total	43668	100.00	91.97	94.17, 72.43
FSC/SSC	36091	82.65	76.01	91.90, 75.21

Region	Count	% Hist	% All	Mean
Total	36091	100.00	76.01	30.71, 548.13
red	27652	76.62	58.24	5.61, 699.88
R8	29	0.08	0.06	206.92, 2825.60
R9	6733	18.66	14.18	6.26, 47.72
green	1677	4.65	3.53	539.75, 15.63

Tuesday, April 17, 2018

Supplementary Figure 12. Gating strategy for sorting of EGFP- or mCherry-positive endothelial cells

Representative scheme of gating strategy for the isolation of EGFP- or mCherry positive endothelial cells from spheroids.

a) Gated have been set on wild type non-labelled cells

b) Sorting of EGFP-positive Wild type endothelial cells cultured as mono-culture

c) Sorting of mCherry-positive *Ccm3*-null endothelial cells cultured as mono-culture

d) Sorting of EGFP-positive Wild type and mCherry-positive *Ccm3*-null endothelial cells harvested from spheroids formed in the co-culture.

Supplementary Figure 13

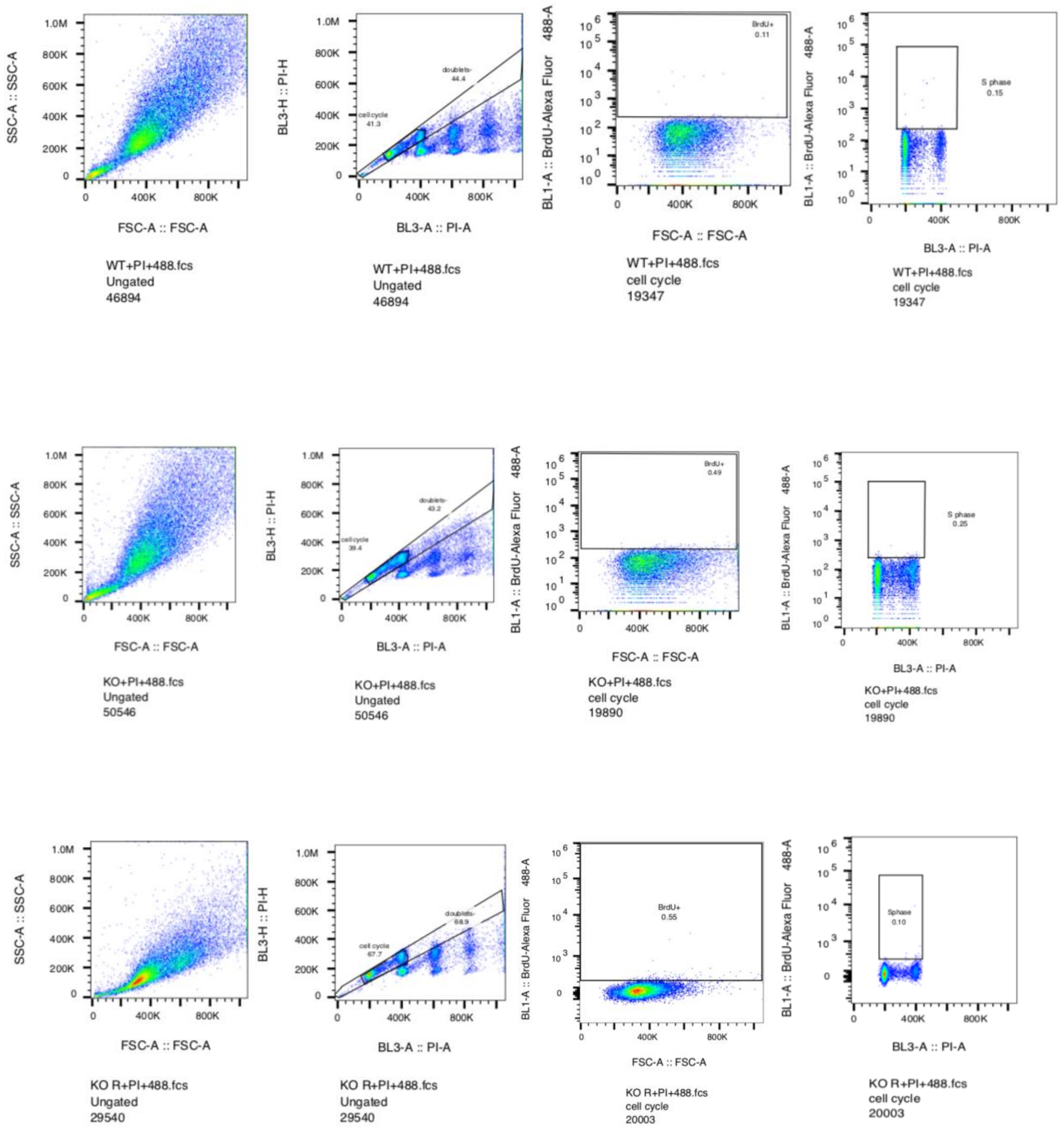

Supplementary Figure 13. Gating strategy for fluorescence-activated-cell-sorting (FACS) of BrdU$^+$ lung-derived immortalized endothelial cells

Representative scheme of gating strategy for the identification of BrdU$^+$ cells to analyze the S-phase of *Ccm3*$^{+/+}$, *Ccm3*$^{-/-}$ and reconstituted *Ccm3*$^{-/-}$ + *hCcm3*-EGFP.

Gates for the sorting of BrdU$^+$ cells have been set on wild-type, knock-out and reconstituted cells labelled with the only 488 - secondary antibody.

Supplementary Figure 14

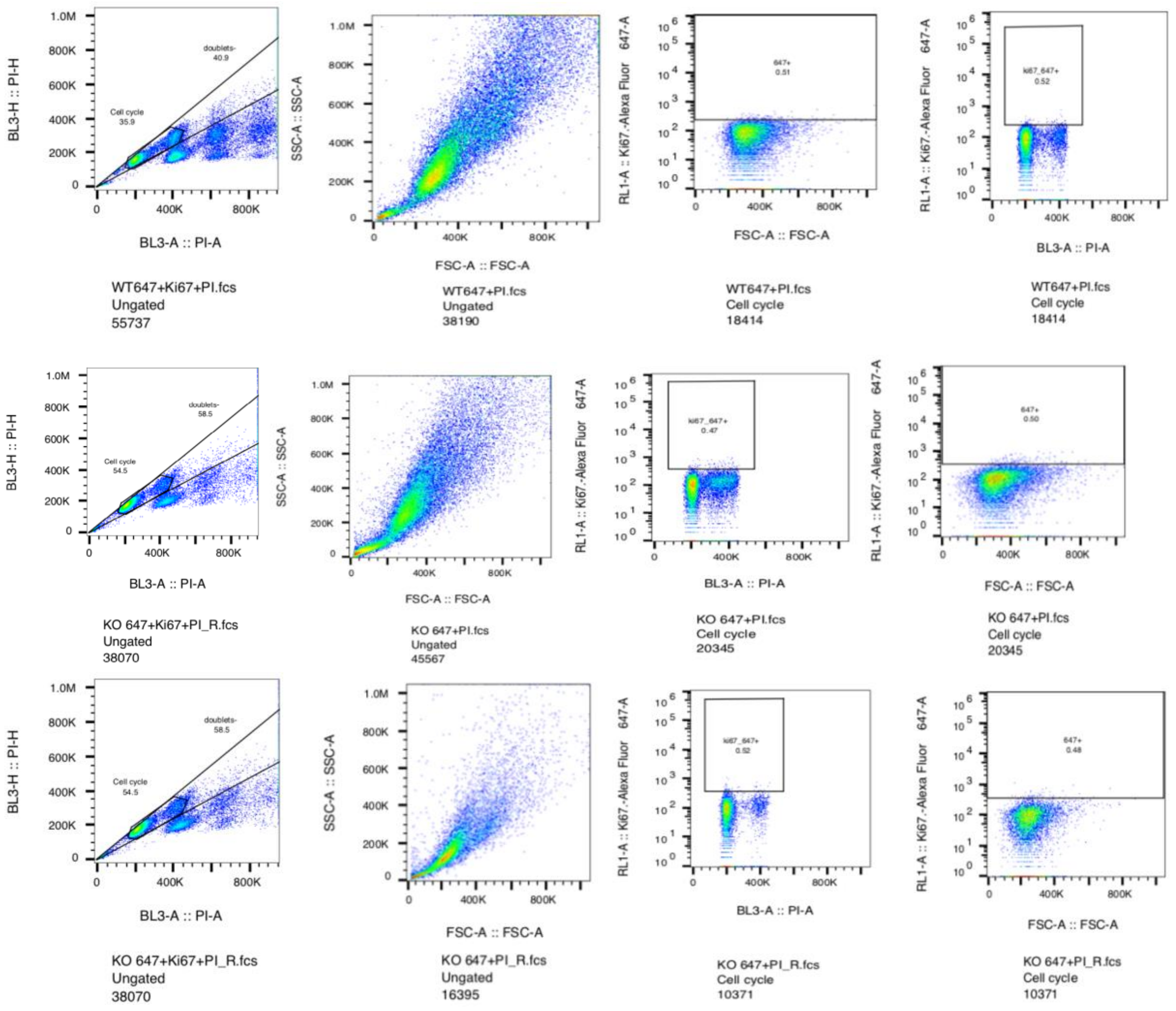

Supplementary Figure 14. Gating strategy for fluorescence-activated-cell-sorting (FACS) of Ki67^{+} lung-derived immortalized endothelial cells.

Representative scheme of gating strategy for the identification of Ki67^{+} cells to analyze the cell cycle of *Ccm3*$^{+/+}$, *Ccm3*$^{-/-}$ and reconstituted *Ccm3*$^{-/-}$ + *hCcm3*-EGFP.

Gates for the sorting of Ki67^{+} cells have been set on wild-type, knock-out and reconstituted cells labelled with the only 647 - secondary antibody.
