# Supplementary material for: Endothelial cell clonal expansion in the development of cerebral cavernous malformations
Source: Nat Commun. 2019 Jun 24;10:2761. doi: 10.1038/s41467-019-10707-x (PMC6591323; doi:10.1038/s41467-019-10707-x)
Supplement: Supplementary file 3 — Description of Additional Supplementary Files [file 41467_2019_10707_MOESM3_ESM.docx]

**Description of Additional Supplementary Files**

**File Name: Supplementary Movie 1**

**Description:** Newly formed lesion 3D reconstruction of a lesion from acute P8 Cdh5(PAC)-Cre-ERT2/Ccm3f/f/R26R-Confetti mouse showing a newly formed lesion. Vessels are stained for Podocalyxin. This movie refers to Figure 1e left panel.

**File Name: Supplementary Movie 2**

**Description:** Small lesion 1 3D reconstruction of a lesion from acute P8 Cdh5(PAC)-Cre-ERT2/Ccm3f/f/R26R-Confetti mouse showing a small lesion. Vessels are stained for Podocalyxin. This movie refers to Figure 1e central panel.

**File Name: Supplementary Movie 3**

**Description:** Small lesion 2 3D reconstruction of a lesion from acute P8 Cdh5(PAC)-Cre-ERT2/Ccm3f/f/R26R-Confetti mouse showing a small lesion. Vessels are stained for Podocalyxin. This movie refers to Figure 1e central panel.

**File Name: Supplementary Movie 4**

**Description:** Small lesion 3 3D reconstruction of a lesion from acute P8 Cdh5(PAC)-Cre-ERT2/Ccm3f/f/R26R-Confetti mouse showing a small lesion. Vessels are stained for Podocalyxin. This movie does not refer to any previously shown figure.

**File Name: Supplementary Movie 5**

**Description:** Large lesion 3D reconstruction of a lesion from acute P8 Cdh5(PAC)-Cre-ERT2/Ccm3f/f/R26R-Confetti mouse showing a large lesion. Vessels are stained for Podocalyxin. This movie refers to Figure 1e right panel.

**File Name: Supplementary Movie 6**

**Description:** Mulberry lesion 3D reconstruction of a lesion from acute P8 Cdh5(PAC)-Cre-ERT2/Ccm3f/f/R26R-Confetti mouse showing a large mulberry lesion. Vessels are stained for Podocalyxin. This movie refers to Figure 1f.

**File Name: Supplementary Movie 7**

**Description:** Chronic P14 cerebellum Images taken from a chronic P14 Cdh5(PAC)-Cre-ERT2/Ccm3f/f/R26R-Confetti cerebellum. (a) Z-projection of a 1 mm thick slice showing the distribution of lesions. (b, c) Magnification and 3D reconstruction of the selected regions. Vessels are stained for Podocalyxin. This movie refers to Figure 2c.

**File Name: Supplementary Movie 8**

**Description:** Large cavernomas from chronic model Images taken from a chronic P14 Cdh5(PAC)-Cre-ERT2/Ccm3f/f/R26R-Confetti cerebellum, showing the multi lumen shape of the cavernomas and their clonality. Vessels are stained for Podocalyxin; L indicates lumen. This movie refers to Figure 3c.

**File Name: Supplementary Movie 9**

**Description:** Time lapse of spheroid formation in vitro Lung immortalised LifeAct-EGFP Ccm3+/+ and LifeAct-mCherry Ccm3-/- cells were mixed as singlecell suspensions (1:1) and plated and cultured under standard conditions. Time-lapse images were acquired as soon as cells reached the confluency. Time is expressed as days : hours : minutes : seconds : milliseconds.

**File Name: Supplementary Movie 10**

**Description:** Time lapse of wound healing assay Lung immortalized Ccm3+/+ cells which expressed LifeAct-EGFP and Ccm3-/- cells which expressed LifeAct-mCherry were seeded in each side of an ibidi silicon insert and grown till confluence. Time-lapse images were acquired immediately after the insert removal. This movie refers to Extended Data Figure 8b. Time is expressed as hours : minutes : seconds : milliseconds.

**File Name: Supplementary Movie 11**

**Description:** Time lapse of wound healing assay after gap closure. Lung immortalized Ccm3+/+ and Ccm3-/- cells which expressed either LifeAct-EGFP or LifeActmCherry were seeded in each side of an ibidi silicon insert and grown till confluence. Time-lapse images were acquired after cells were allowed to close the wound. Left panel: LifeAct-EGFP and LifeAct-mCherry expressing Ccm3+/+ cells. Right panel: LifeAct-EGFP Ccm3+/+ and LifeAct-mCherry Ccm3-/- cells. This movie refers to Extended Data Figure 8c, d. Time is expressed as hours : minutes : seconds : milliseconds.
