# Supplementary material for: Endothelial cell clonal expansion in the development of cerebral cavernous malformations
Source: Nat Commun. 2019 Jun 24;10:2761. doi: 10.1038/s41467-019-10707-x (PMC6591323; doi:10.1038/s41467-019-10707-x)
Supplement: Supplementary file 15 — Reporting Summary [file 41467_2019_10707_MOESM15_ESM.pdf]

nature research

Corresponding author(s): Matteo Malinverno
Elisabetta Dejana

Last updated by author(s): May 27, 2019

Reporting Summary

Nature Research wishes to improve the reproducibility of the work that we publish. This form provides structure for consistency and transparency in reporting. For further information on Nature Research policies, see Authors & Referees and the Editorial Policy Checklist.

Statistics

For all statistical analyses, confirm that the following items are present in the figure legend, table legend, main text, or Methods section.

n/a	Confirmed	
☐	☒	The exact sample size (*n*) for each experimental group/condition, given as a discrete number and unit of measurement
☐	☒	A statement on whether measurements were taken from distinct samples or whether the same sample was measured repeatedly
☐	☒	The statistical test(s) used AND whether they are one- or two-sided<br>*Only common tests should be described solely by name; describe more complex techniques in the Methods section.*
☐	☒	A description of all covariates tested
☒	☐	A description of any assumptions or corrections, such as tests of normality and adjustment for multiple comparisons
☐	☒	A full description of the statistical parameters including central tendency (e.g. means) or other basic estimates (e.g. regression coefficient) AND variation (e.g. standard deviation) or associated estimates of uncertainty (e.g. confidence intervals)
☐	☒	For null hypothesis testing, the test statistic (e.g. *F*, *t*, *r*) with confidence intervals, effect sizes, degrees of freedom and *P* value noted<br>*Give P values as exact values whenever suitable.*
☒	☐	For Bayesian analysis, information on the choice of priors and Markov chain Monte Carlo settings
☒	☐	For hierarchical and complex designs, identification of the appropriate level for tests and full reporting of outcomes
☒	☐	Estimates of effect sizes (e.g. Cohen's *d*, Pearson's *r*), indicating how they were calculated

Our web collection on statistics for biologists contains articles on many of the points above.

Software and code

Policy information about availability of computer code

Data collection	Microsoft Excell for Mac version 15.34;
Data analysis	Microsoft Excell for Mac version 15.34; Prism 7; ImageJ; Arivis Vision4D

For manuscripts utilizing custom algorithms or software that are central to the research but not yet described in published literature, software must be made available to editors/reviewers. We strongly encourage code deposition in a community repository (e.g. GitHub). See the Nature Research guidelines for submitting code & software for further information.

Data

Policy information about availability of data

All manuscripts must include a data availability statement. This statement should provide the following information, where applicable:

- Accession codes, unique identifiers, or web links for publicly available datasets
- A list of figures that have associated raw data
- A description of any restrictions on data availability

All the data supporting the findings of this study are available from the corresponding authors upon reasonable request.

Field-specific reporting

Please select the one below that is the best fit for your research. If you are not sure, read the appropriate sections before making your selection.

☒ Life sciences ☐ Behavioural & social sciences ☐ Ecological, evolutionary & environmental sciences

For a reference copy of the document with all sections, see nature.com/documents/nr-reporting-summary-flat.pdf

Life sciences study design

All studies must disclose on these points even when the disclosure is negative.

Sample size	Each experiment has been repeated at least three independent times; this is usually enough to study effect sizes of 2 fold. When higher variability was expected, for in vivo experiments for instance, the sample size was increased.
Data exclusions	Every experiments contained positive controls. Only when the positive controls did not work, the entire experiment were excluded by the analysis.
Replication	In vivo experiments were replicated at different times on different litters, coming from different breeding pairs. In vitro experiments were always replicated with cells at different passage number and in different days.
Randomization	For in vivo experiments, animals were randomly included to the Control or treated group.
Blinding	All the analysis were performed by a blinded operator.

Reporting for specific materials, systems and methods

We require information from authors about some types of materials, experimental systems and methods used in many studies. Here, indicate whether each material, system or method listed is relevant to your study. If you are not sure if a list item applies to your research, read the appropriate section before selecting a response.

Materials & experimental systems

n/a	Involved in the study	
☐	☒	Antibodies
☐	☒	Eukaryotic cell lines
☒	☐	Palaeontology
☐	☒	Animals and other organisms
☒	☐	Human research participants
☒	☐	Clinical data

Methods

n/a	Involved in the study	
☒	☐	ChIP-seq
☐	☒	Flow cytometry
☒	☐	MRI-based neuroimaging

Antibodies

Antibodies used	The following primary antibodies were used: Cd31 APC-conjugated anti-PECAM-1 (551262, BD Pharmigen), pSMAD1/5 rabbit (9516, Cell Signalling), SMAD1 rabbit (9743, Cell Signalling), SMAD3 rabbit (9523, Cell Signalling); Vinculin mouse (V9264, Sigma-Aldrich); Fsp1 rabbit (07-2274, Merck Millipore), Cd31 (2H8, armenian hamster; Merck Millipore), Cd31 rat (MEC 13.3, BD Biosciences), pSMAD3 rabbit (ab52903, AbCam), Fn1 rabbit (ab23750, AbCam), Sca1 rat (ab51317, AbCam), Id1 rabbit (sc-488, Santa Cruz), Erg1/2/3 rabbit (sc-353, Santa Cruz Biotechnology), Podocalyxin goat (AF 1556, R&D Systems), Klf4 goat (AF3158, R&D Systems), Collagen IV rabbit (2150-1470, Bio-Rad), GFP rabbit (A-6455, ThermoFisher), alpha-SMA Cy3-conjugated mouse (C6198, Sigma-Aldrich), rat anti-Ki67 (clone SolA15, Invitrogen), purified mouse anti-BrdU (347580, BDbioscience), rabbit anti-PPH3 Ser10 (06-570, Millipore).
Validation	Validation has been done by incubating samples with either the primary Ab or vehicle followed by detection with proper secondary antibody

Eukaryotic cell lines

Policy information about cell lines

Cell line source(s)	Lung immortalised endothelial cells isolated from ccm3 floxed mice and recombined i vitro, as described in Bravi et al. PNAS 2015
Authentication	Not authenticated
Mycoplasma contamination	All cell lines were micoplasma free
Commonly misidentified lines (See ICLAC register)	*Name any commonly misidentified cell lines used in the study and provide a rationale for their use.*

Animals and other organisms

Policy information about studies involving animals; ARRIVE guidelines recommended for reporting animal research

Laboratory animals	The Cdh5(PAC)-Cre-ERT2/Ccm3fl/f , Cdh5(PAC)-Cre-ERT2/Ccm3fl/fl/R26-EYFP and Cdh5(PAC)-Cre-ERT2/Ccm3fl/fl/R26R-Confetti mice were on a pure C57/BL6J background, while the ProcrCreERT2-IRES-tdTomato/+ on mixed one.
Wild animals	The study did not involve wild animals
Field-collected samples	The study did not involve samples collected from the field
Ethics oversight	All of the procedures with the mice were performed in agreement with the Institutional Animal Care and Use Committee (IACUC) of FIRC Institute of Molecular Oncology, in compliance with the guidelines established in the Principles of Laboratory Animal Care (Directive 86/609/EEC) and approved by the Italian Ministry of Health.

Note that full information on the approval of the study protocol must also be provided in the manuscript.

Flow Cytometry

Plots

Confirm that:

☒ The axis labels state the marker and fluorochrome used (e.g. CD4-FITC).

☒ The axis scales are clearly visible. Include numbers along axes only for bottom left plot of group (a 'group' is an analysis of identical markers).

☒ All plots are contour plots with outliers or pseudocolor plots.

☒ A numerical value for number of cells or percentage (with statistics) is provided.

Methodology

Sample preparation	Endothelial cells were isolated from brains using a dissociator (gentleMACS; 130-093-235; Miltenyi) and the Adult Brain Dissociation kits (130-107-677; Miltenyi), following the manufacturer protocol. After dissociation, the cells were re-suspended in PBS with 2% foetal bovine serum and 2 mM EDTA, and incubated for 30 min with an APC-conjugated anti-Cd31 antibody, for FACS sorting.<br>For cell isolation from spheroids, the spheroids were removed from the monolayer following gentle shaking of the plate, and collected and trypsinised. The single-cell suspensions obtained were immediately sorted by FACS for EGFP and mCherry expression.<br>For FACS analysis , cultured cells were collected after trypsinisation and processed as described in Methods section.
Instrument	MoFlo Astrios EQ by Beckman Coulter 4 lasers (405, 488, 561, 640). Attune NxT Life Technologies.
Software	Summit 6.3
Cell population abundance	For cells isolated from brains, Cd31-positive endothelial cells after gating were around 9%.<br>For cells isolated from spheroids, after gating: EGFP-positive cells from WT mono-culture were around 90%; mCherry-positive cells from Ccm3-null mono-culture were around 90%; from the spheroids Ccm3-null were 75% and WT 5 % of total cells.
Gating strategy	Gating strategies are described in details in Supplementary Information.

☒ Tick this box to confirm that a figure exemplifying the gating strategy is provided in the Supplementary Information.
